# The role of the immune microenvironment in bone, cartilage, and soft tissue regeneration: from mechanism to therapeutic opportunity

**DOI:** 10.1186/s40779-022-00426-8

**Published:** 2022-11-19

**Authors:** Yuan Xiong, Bo-Bin Mi, Ze Lin, Yi-Qiang Hu, Le Yu, Kang-Kang Zha, Adriana C. Panayi, Tao Yu, Lang Chen, Zhen-Ping Liu, Anish Patel, Qian Feng, Shuan-Hu Zhou, Guo-Hui Liu

**Affiliations:** 1 Department of Orthopedics, Union Hospital, Tongji Medical College, Huazhong University of Science and Technology, Wuhan, 430022 China; 2 Hubei Province Key Laboratory of Oral and Maxillofacial Development and Regeneration, Wuhan, 430022 China; 3 Department of Chemical and Biomolecular Engineering, Ohio University, Athens, OH 45701 USA; 4 Key Laboratory of Biorheological Science and Technology,Ministry of Education College of Bioengineering, Chongqing University, Shapingba, Chongqing, 400044 China; 5 Department of Plastic Surgery, Brigham and Women’s Hospital, Harvard Medical School, Boston, MA 02152 USA; 6 Department of Orthopaedics, Ruijin Hospital, Shanghai Jiao Tong University School of Medicine, Shanghai, 200025 China; 7 Department of Physics, Center for Hybrid Nanostructure (CHyN), University of Hamburg, Hamburg, 22761 Germany; 8 Joint Laboratory of Optofluidic Technology and System,National Center for International Research on Green Optoelectronics, South China Academy of Advanced Optoelectronics, South China Normal University, Guangzhou, 510006 China; 9 Skeletal Biology Laboratory, Department of Orthopedic Surgery, Brigham and Women’s Hospital, Harvard Medical School, Boston, MA 02120 USA; 10 Harvard Stem Cell Institute, Harvard University, Cambridge, MA 02138 USA

**Keywords:** Immune microenvironment, Regeneration, Cell-cell interaction, Tissue engineering, Biomaterials

## Abstract

Bone, cartilage, and soft tissue regeneration is a complex spatiotemporal process recruiting a variety of cell types, whose activity and interplay must be precisely mediated for effective healing post-injury. Although extensive strides have been made in the understanding of the immune microenvironment processes governing bone, cartilage, and soft tissue regeneration, effective clinical translation of these mechanisms remains a challenge. Regulation of the immune microenvironment is increasingly becoming a favorable target for bone, cartilage, and soft tissue regeneration; therefore, an in-depth understanding of the communication between immune cells and functional tissue cells would be valuable. Herein, we review the regulatory role of the immune microenvironment in the promotion and maintenance of stem cell states in the context of bone, cartilage, and soft tissue repair and regeneration. We discuss the roles of various immune cell subsets in bone, cartilage, and soft tissue repair and regeneration processes and introduce novel strategies, for example, biomaterial-targeting of immune cell activity, aimed at regulating healing. Understanding the mechanisms of the crosstalk between the immune microenvironment and regeneration pathways may shed light on new therapeutic opportunities for enhancing bone, cartilage, and soft tissue regeneration through regulation of the immune microenvironment.

## Background

Bone, cartilage, and soft tissue injury are common clinical conditions that can severely impair function and limit the quality of life [1–3]. Although the field of tissue engineering has rapidly developed, thus providing promising means of adequately repairing tissue damage, soft tissue defects, particularly large-area tissue injuries or complex injuries involving multiple tissues, remain major challenges for clinicians [4, 5]. Regenerative medicine is an important branch of translational medicine in bioengineering and cellular biology that involves the replacement, reconstruction, or regeneration of cells, tissues, or organs. The purpose is to stimulate the body’s inherent repair mechanisms to effectively cure injured tissue [6]. Bone, cartilage, and soft tissue regeneration is a dynamically balanced process involving the metabolism, differentiation, and migration of tissue cells, including complex interactions between the immune and musculoskeletal systems [7, 8].

Immune cells are involved in the regulation of tissue homeostasis; in contrast, tissue cells influence the survival and function of immune cells; as such, immunomodulation plays a critical role in tissue repair and regeneration [9, 10]. The inflammatory response is important in maintaining tissue homeostasis and has dual roles in this regulation process [11]. It serves as a protective response in the promotion of tissue regeneration and is often a major cause of tissue damage in infectious diseases, immunologic alteration, and trauma [12–14]. When the inflammatory response is activated by injury, abundant cells derived from peripheral blood monocytes are present in tissues, and the specific immune microenvironment drives these cells’ sequence of repair [15].

The immune microenvironment plays an important role in the healing, repair, and regeneration of tissues, and can be reshaped by intrinsic and exoteric factors, such as stem cells [16]. Currently, the major roles of stem cells in the regulation of the immune microenvironment and the connection between stem cells and tissue regeneration has been well studied [17]. The reparative functions of mesenchymal stem cells (MSCs) in a wide array of inflammatory diseases rely on their immunomodulation and the release of various bioactive cytokines [18]. Specifically, tissue regeneration is closely associated with the immune microenvironment surrounding the injured tissue. The immunoregulatory function of MSCs affects the clinical application and translation of MSC-based regenerative therapy [19].

Stem cells exert their immunomodulatory roles by producing various regulatory cytokines, such as interleukin (IL)-4, IL-7, IL-10, interferon-γ (IFN-γ), and prostaglandin E2 (PGE2) [20]. Interestingly, emerging evidence indicates that small extracellular vesicles (sEVs) secreted in a paracrine manner are an important means through which stem cells regulate the immune microenvironment [21]. For example, MSC-derived sEVs significantly ameliorate the development of autoimmune and neurodegenerative disorders by re-programming the immune environment under pathological conditions [22]. sEVs are versatile membrane vesicles with a regulatory function through delivery of various bioactive molecules throughout the intercellular microenvironment. sEVs mediate the crosstalk between cells and modulate the immune microenvironment in a paracrine manner [10]. Evidence has indicated a correlation between sEVs and stem cells, thus suggesting their high potential for promoting cellular proliferation and migration to the injured tissue [10, 13]. Moreover, stem cell-derived sEVs facilitate tissue regeneration through modulation of the immune microenvironment [13]. However, stem cells and sEVs also have substantial disadvantages in clinical application, such as complex components, unstable biological activity, low targeting, and difficulty in preservation [23, 24]. Therefore, interest has increased in developing new strategies to maximize the therapeutic effects of stem cells and EVs.

Currently, various tissue-specific biomaterials with cytokines and immunomodulatory effects promoting tissue regeneration have been developed and implanted into sites of damaged tissue to enhance the therapeutic efficacy of tissue regeneration. The biomaterial-based strategy can provide physical support to transplanted cells, and stem cells can rapidly proliferate and differentiate to compensate for the lost tissue cells [25]. Furthermore, the therapeutic effects can be enhanced if biomaterials exert immunomodulatory effects and inhibit the local overactivated immune responses [26]. The properties of the biomaterial for tissue regeneration vary depending on the target damaged tissue, and the immunosuppression provided by biomaterials has prominent effects on tissue repair and regeneration [27].

The role of the immune microenvironment in regulating tissue regeneration has attracted attention [28–32]. For example, Yang et al. [28] have summarized the roles of multiple immune cells and immune cytokines in bone regeneration. Similarly, a recent study has systematically introduced the new developments in cellular crosstalk between immune cells and stem cells, and provided advanced insights into the application of biomaterial-based strategies in the promotion of tissue regeneration [32]. However, few studies have comprehensively summarized the regulatory roles of stem cells and the immune microenvironment, and how to balance these roles through immunomodulatory biomaterials in bone, cartilage, and soft tissue regeneration. Therefore, we will discuss the role of the immune microenvironment in tissue regeneration, focusing on stem cells and immune cells, to discuss the immune mechanisms in the tissue repair and regeneration processes, and shed light on promoting the curative effects of treatments for severe tissue injury.

## Stem cell signals in the regulation of tissue regeneration

Stem cells are a critical primitive cell type with differentiation and regeneration potential. Tissue regeneration is coordinated by stem cells, which not only compensate for lost functional cells but also exert self-renewal functions [18, 21]. Stem cells reside in damaged tissues and are the cellular source of the regeneration process [19]. In response to tissue damage, stem cells accelerate the production of specific types of differentiated cells, thus promoting tissue regeneration. Classic stem cell signals are often activated within tissue cells, and consequently enable damaged tissues to self-renew and proliferate [30]. Thus, stem cell signals play a major role in tissue regeneration, and stem cell-based therapies are a prominent trend in regenerative medicine.

### Stem cells and bone regeneration

Bone regeneration is an intricate and highly orchestrated biological regulatory process involving different cell types and their activated signaling pathways [32, 33]. Stem cells, particularly skeletal stem cells or MSCs, engage in bone regeneration, owing to their self-renewal and differentiation ability, secretion of active cytokines, and modulation of other cells in host tissues [34]. The involvement of MSCs in bone regeneration processes is mediated by active molecules, such as hormones and growth factors, and their stimulated cellular networks [33] (Table 1).

**Table 1:** Main signaling pathways in bone regeneration

Signaling pathways	Major characteristics and functions	Applications in bone regeneration
BMP-2	BMP-2 initially binds to type II receptors on the cell membrane and then binds to type I receptors to form a dimer. The activated type I receptors rapidly phosphorylate the serine residues of SMAD-1, SMAD-5, and SMAD-8, and the activated SMADs are transferred into the nucleus and exert biological effects [35, 36]	The osteoporotic phenotype was reversed in mice with systematic injections of rhBMP-2 [35] MSCs infected with a recombinant adenoviral vector encoding human BMP-2 were capable of repairing bone defects in ectopic sites through engrafting and forming bone and cartilage in mice [36, 37] 3D bioprinted implants containing a VEGF gradient, paired with spatially defined BMP-2 localization and release kinetics, expedited the healing of defects in large bone with the minuscule formation of heterotopic bone [38] mRNA-based BMP-2 therapy was used to facilitate bone regeneration in mice [39–41]
NGF-p75 signaling	Cranial bone injuries stimulate NGF expression and its signals via p75 in resident osteogenic precursors that affect their migration into the damaged tissue and promote bone regeneration [42]	NGF-p75 signaling pathway coordinates skeletal cell migration during early bone repair [42]
FAK	Mechanotransduction via the FAK signaling pathway in skeletal stem cells promotes stem-cell-mediated regeneration of adult skeletal tissue [43, 44]	Inhibiting FAK abolishes bone regeneration in distraction osteogenesis [44]
NF-κB	Activation of NF-κB signaling in osteoclasts is crucial for their differentiation and activation, whereas the activation in osteoblasts inhibits bone formation. These unique characteristics imply the great potential of NF-κB as a therapeutic target for bone disorders and regeneration [45]	The activating NF-κB signaling may be one of the extrinsic mechanisms by which skeletal stem cell function decline during human skeletal aging [45–49]

*BMP-2* bone morphogenetic protein-2, *FAK* focal adhesion kinase, *NF-κB* nuclear factor-κB, *NGF* nerve growth factor, *VEGF* vascular endothelial growth factor, *rhBMP-2* recombinant human bone morphogenetic protein-2, *MSCs* mesenchymal stem cells

Bone morphogenetic protein-2 (BMP-2) is a signaling molecule with critical roles in bone regeneration. Recombinant human BMP-2 (rhBMP-2) has been widely used in clinical settings to enhance bone regeneration. Our previous research has indicated that the osteoporotic phenotype is reversed in mice with systematic injections of rhBMP-2. rhBMP-2 injection enhances the osteogenic activity of MSCs, thus suggesting that rhBMP-2 and other active anabolic compounds are effective in targeting MSCs [35]. Through a skeletal gene therapy approach, we have found that MSCs infected with a recombinant adenoviral vector encoding human BMP-2 can repair bone defects in ectopic sites through engrafting and forming bone and cartilage in mice [36, 37]. Freeman et al. [38] have reported that 3D bioprinted implants containing a vascular endothelial growth factor (VEGF) gradient, paired with spatially defined BMP-2 localization and release kinetics, expedites the healing of defects in large bone with minimal formation of heterotopic bone. A major advancement in the field of bone regeneration has been the development of mRNA-based BMP-2 therapy. For example, to avoid the high costs and adverse effects of rhBMP-2, such as inflammatory complications, ectopic bone formation, and tumor formation [39], De La Vega et al. [40] have reported that using a modified mRNA encoding BMP-2 is another approach to bone regeneration. This novel approach, compared with the recombinant protein approach, results in significantly better healing of large, critically sized, segmental osseous defects of long bones and has no adverse effects, possibly because of its transient and anatomically restricted expression of BMP-2 [40]

In recent years, several high-impact studies have reported the critical intracellular signals in bone regeneration. De Simone et al. [41] reported that rhythmic traveling waves of extracellular signal-regulated kinase activity modulate the growth of bone temporally and spatially in regenerating zebrafish. After injury, inflammatory signals cause bone regeneration to commence simultaneously with infiltration by sensory nerve fibers. Xu et al. [42] demonstrated that cranial bone injuries stimulate nerve growth factor expression and signaling via p75 in resident osteogenic precursors that affect their migration into the damaged tissue, thus suggesting that nerve growth factor-p75 signaling has potential roles in bone regeneration. Ambrosi et al. [43] reported that intrinsic skeletal stem cell aging in mice alters signaling in the bone marrow niche to a degenerative inflammatory niche, thus leading to poorly regenerated bones because of fragility. BMP-2 has been used to activate skeletal stem cells together with a CSF1 antagonist to inhibit bone resorption, thus eliciting youthful bone regeneration in older bone [43]. Using chromatin and transcriptional profiling, Ransom et al. [44] demonstrated that mechanotransduction via the focal adhesion kinase signaling pathway in skeletal stem cells promotes stem cell-mediated regeneration of adult skeletal tissue. The dedifferentiation of mature cells is a cellular process strongly associated with tissue regeneration. Osteoblasts dedifferentiate into osteogenic progenitors during zebrafish fin regeneration, thus providing source cells for bone restoration [45]. Through in vivo chemical identification of mediators of osteoblast dedifferentiation and fin regeneration, Mishra et al. [45] have found that the NF-κB pathway is active in mature osteoblasts and is downregulated before dedifferentiation. In contrast, inhibition of NF-κB signaling has been found to enhance dedifferentiation, thus clarifying the molecular regulation of regenerative cellular plasticity [45]

In summary, although debates remain regarding the origins, functions, developmental potential, and possible therapeutic uses of MSCs [46]—mainly because MSCs are commonly defined by their in vitro functions, whereas their functions in vivo are insufficiently defined [47]—these stem cells and the signaling molecules associated with their proliferation, migration, and differentiation are critical in bone homeostasis and regeneration [48, 49]

### Stem cell signals in the regulation of cartilage regeneration

Cartilage is mainly composed of collagen and proteoglycans. As an avascular and aneural tissue, cartilage lacks self-healing ability after damage, which can be triggered by trauma, aging, obesity, immune diseases, tumor resection, and osteoarthritis (OA). After injury is initiated in cartilage, the two opposing bones rub against each other, and joint replacement is eventually required in the absence of early intervention. Since the 1930s, clinical interventions for cartilage lesions, including surgical and non-surgical approaches, have evolved from palliative to reparative and most recently to regenerative strategies [50]. However, the complex bi-phasic structure of the osteochondral unit and the relatively low metabolic activity of chondrocytes in articular cartilage substantially hinder repair. Cartilage is remodeled dynamically by signaling pathways that are controlled by cells and the extracellular matrix (ECM) [51]. Using cell signals has therefore provided a longer tether for the regenerative management of cartilage injury. Chondrocytes are one of the primary choices in cartilage regeneration, because they are the prominent resident cell type in articular cartilage. However, their application is largely limited by their inferior isolation efficiency, low proliferation rate, and high possibility of dedifferentiation into fibroblasts during expansion [52, 53]. Hence, the use of stem cells has gained momentum in the field, owing to their ability to proliferate and directionally differentiate into chondrocytes. Preclinical and clinical studies involving stem cells have demonstrated significantly better outcomes with cartilage regeneration than with traditional cell-free strategies [54, 55].

Stem cells used for the restoration of cartilage defects can be classified into three main categories: adult stem cells (ASCs), embryonic stem cells (ESCs), and induced pluripotent stem cells (iPSCs) [56]. The most widely used form of stem cells is ASCs, which are found in adult body tissues; these cells include skeletal stem cells and MSCs, adipose tissue MSCs, joint synovium MSCs, and peripheral blood MSCs. These cells have benefits of the relative ease of isolation and greater availability than ESCs and iPSCs, which are found in mammalian embryos and genetically reprogrammed somatic cells, respectively [57, 58]. The age-associated decline in proliferation, and the association with hypertrophic cartilage or fibrocartilage formation limit the application of ASCs in cartilage regeneration. ESCs are considered the most suitable type for articular cartilage regeneration, because they can indefinitely self-renew and can be directed to differentiate into both lineages of bone and cartilage, owing to their pluripotency [55, 59]. The disadvantages of using ESCs in clinical practice include the difficulty in obtaining functional chondrocytes from human ESCs and ethical issues [60]. The discovery of iPSCs in 2006 opened a gateway for cartilage regeneration, because these cells possess pluripotency and the potential for self-renewal, similarly to ESCs but without ethical concerns [60, 61]. However, standard and simple protocols to guide iPSCs toward chondrogenic differentiation are lacking, and these cells’ effectiveness in hyaline cartilage production is highly reliant on environmental cues [62–66]. For example, Lee et al. [62] have found that chondrocytes derived from iPSCs through mesodermal and ecto-mesodermal differentiation have distinct activities and functions. Similar to the search for better scaffold design and effective biological stimulation, appropriate stem cell selection remains a challenge for functioning cartilage regeneration.

In terms of the therapeutic mechanisms involving stem cells in cartilage regeneration, cartilage tissue restoration was previously believed to be achieved via the directional chondrogenic differentiation of implanted stem cells triggered by the surrounding microenvironment (differentiation theory) [67–69]. However, recent evidence suggests that exogenous (donor) stem cells do not directly contribute to the formation of regenerated cartilage tissue by turning into chondrocytes [70]. Instead, they regulate the microenvironment of the defect area by producing various derivatives, including growth factors, extracellular vesicles (EVs), and ECM, which can alter the fate of host cells such as endogenous (host) stem cells, chondrocytes, and macrophages (paracrine effect) [71, 72]. For example, these paracrine signaling pathways induce the homing and proliferation of resident chondrocytes, promote the chondrogenic differentiation of endogenous stem cells, and positively modulate the anti-inflammatory process by influencing host macrophages to facilitate cartilage regeneration [70, 71, 73–75].

Although the detailed mechanisms underlying stem cell-regulated cartilage regeneration remain unclear, the patterning, growth, maturation, and homeostasis of cartilage tissue are known to be exquisitely tuned by a series of signaling pathways, which govern the fate of stem cells [76, 77]. Consequently, a better understanding of these signaling pathways should offer therapeutic opportunities for cartilage regeneration. In Table 2, we summarize the crucial signaling pathways that are responsive to cartilage functional behavior, along with recent examples of use of these signals in stem cell-mediated cartilage regeneration; the mechanisms underlying each of these signals have been investigated in different microenvironment cues. Healthy cartilage function is finely tuned through synergistic functions of multiple signaling pathways, and extensive crosstalk exists among these pathways to maintain a dynamic balance between synthetic and catabolic activities of cartilage tissue.

**Table 2:** Main signaling pathways in cartilage regeneration

Signaling pathways	Major characteristics and functions	Applications in bone regeneration
Notch	Notch signaling contributes to vertebrate development [78]. Its activation and expression exhibit a fluctuant pattern throughout chondrocyte maturation and display a dual function: sustained Notch activation in joint cartilage leads to an OA-like pathology, while transient activation could achieve increased cartilage ECM synthesis and successful joint maintenance [79, 80]	Intra-articular injection of BMP9 transfected AT-MSCs enhanced type II collagen and aggrecan expression and promoted cartilage repair through Notch1/Jagged1 signaling pathway in mouse knee OA model [81] Micro ribonucleic acid (miR9) promoted the differentiation of chondrocyte and cartilage regeneration in the rabbit OA model by mediating type II collagen expression via the down-regulation of the Notch signaling pathway [82]
Wnt	Wnt signaling contributes to skeletal development and growth [83]. Wnts and MITF signaling pathways are responsible for the generation of off-target differentiation into neural cells and melanocytes during chondrogenesis of iPSCs [84]	A synthetic Wnt5a (non-canonical) mimetic ligand, Foxy5 peptide, was conjugated to hyaluronic acid hydrogel to enhance chondrogenic expression of MSCs and cartilage regeneration in subcutaneous pockets on the back of mouse [85] Down-regulation of the Wnt transduction produced better quality articular cartilage-like tissue by MSCs cultured in faster degrading and soft matrices, which were implanted in subcutaneous pockets of mice [86] Injection of SM04690 upregulated Wnt16 expression and therefore ameliorated abnormal subchondral bone remodeling in both rabbit and rat models and enhanced chondrogenesis of fibrocartilage stem cells [87]
TGF-β/BMP	The TGF-β superfamily is consisted of over 40 members that are classified into TGF-β and BMP subfamilies [88]. They play crucial roles in regulating condensation, cell survival and differentiation [89]	Autogenous platelet-rich-plasma treated BM-MSCs-seeded collagen scaffolds indicated accelerated cartilage regeneration through TGF/SMAD pathway in rabbits [90] Naringin-treated BM-MSCs demonstrated an efficient articular cartilage repair in rabbit knees through activation and continuous regulation of the TGF-β superfamily pathways [91] TGF-β1 transfected BM-MSCs seeded in calcium alginate gel improved the repair of rat cartilage defect through canonical SMAD2/3 signaling pathway and inhibited chondrocyte hypertrophy by decreasing hypertrophy makers expression via Hippo pathway [92]
NF-κB	Transcription factor NF-κB is a master regulator of inflammation involved in the pathogenesis and progression of OA [93]. In its inactive state, NF-κB presents in the cytoplasm as a heterotrimer complex and is prevented from entering nuclei; while chondrocytes are stimulated, NF-κB can be released and activated from IκB through degradation and enter nuclei [94]. Therefore, stopping the degradation of IκB could be an effective way to inhibit the NF-κB signaling pathway	Intra-articular injection of IGF-1 prevented the expression of MMPs and various apoptotic markers by inhibiting the NF-κB signaling and suppression of ROS production during OA pathogen and resulted in a better cartilage defect therapy in a rabbit knee OA model [95] Bone defect was treated with anti-osteogenic reagents Fulvestrant and IL1β to inhibit ossification during cartilage regeneration by activating NF-κB signaling [96]
HIF	Articular cartilage survives in a microenvironment devoid of oxygen, which is controlled by HIF signaling [97, 98]. Overexpressing of HIF is clinically associated with OA [99]	3D-printed bioactive ceramic scaffolds containing Sr/Si or Li/Si or Cu ions stimulated cartilage and subchondral bone regeneration in rabbits by promoting chondrocytes maturation via activating HIF pathways [100–102] IOX2, a HIF-1α prolyl hydroxylase domain inhibitor, was used to promote the proliferation and migration of BM-MSCs via stabilizing HIF-1α pathway and accelerating cartilage fracture healing in rats [103]
FGF	FGF signaling accelerates the termination rate of hypertrophic differentiation to help chondrocytes remain within cartilage rather than undergoing hypertrophic maturation prior to ossification [77]. It acts as an antagonist of BMP signaling, which hinders the termination of hypertrophic differentiation [104]	Pellets of MSCs cultured with FGF were implanted into osteochondral defects of mice to promote cartilage regeneration [105] Sulfated alginate hydrogel was used to drive mitogenicity of chondrocytes, promote cartilage matrix production, and prevent chondrocyte dedifferentiation via the mediation of FGF signaling in a heparin-mimetic manner [106, 107]
IGF	IGF can influence metabolic and proliferative processes of cartilage and play a role in protection against ECM degradation [108, 109]. It regulates cartilage growth and homeostasis in TMJ fibrocartilage stem cells [110]	Platelet-derived biomaterial significantly suppressed OA-like pathophysiological characteristics by restoration levels of IGF-1 signaling pathway proteins [111] Combined usage of IGF-1 and TGF-β1 in BM-MSCs-seeded laminin scaffolds enhanced the restoration of hyaline cartilage in a rabbit knee osteochondral defect model [112]

*AT-MSCs* adipose tissue derived-MSCs, *BM-MSCs* bone marrow derived-MSCs, *ECM* extracellular matrix, *FGF* fibroblast growth factor, *IGF* insulin-like growth factor, *iPSCs* induced pluripotent stem cell, *TGF-β* transforming growth factor-β, *TGF-β1* transforming growth factor-β1, *TMJ* temporomandibular joint, *OA* osteoarthritis, *MITF* microphthalmia transcription factor, *BMP* bone morphogenetic protein, *ROS* reactive oxygen species, *MMPs* matrix metalloproteinases

### The correlation between stem cells and soft tissue regeneration

Soft tissue defects, such as those following trauma, tumor resection, and infection, are common clinical encounters, and adequate tissue regeneration is a substantial challenge. Stem cell-based therapy has gained momentum in regenerative medicine [113, 114]. Stem cells modulate tissue metabolism and regeneration mainly via two unique abilities: 1) the ability to self-renew with symmetric division, and 2) the ability to multi-directionally differentiate with asymmetric division [13] (Table 3).

c-Jun N-terminal kinase (JNK) signaling is among the most important regulatory signals in soft tissue regeneration; JNK regulates the activity of stem cells involved in soft tissue repair and regeneration [115]. JNKs are important molecules mediating the intracellular responses of stem cells to many different types of stimuli in the external cellular microenvironment [116]. JNK function is essential for achieving a delicate balance between cell death and stem cell survival to promote soft tissue repair, remodeling, and regeneration [117]. Dhoke et al. [118] have reported that transplantation of preconditioned stem cells enhances soft tissue regeneration with a robust antioxidant defensive mechanism through activation of JNK signaling. Similarly, Jiang et al. [119] have found that JNK signaling plays a critical role in regulating the differentiation of MSCs into keratinocytes and promotes tissue regeneration. An in-depth understanding of the mechanism underlying how JNK signaling mediates soft tissue regeneration would aid in the development of new effective therapies.

Epithelial regeneration is a crucial component of soft tissue regeneration, and an in-depth understanding of the regulatory roles of ESCs and their effects on tissue homeostasis might elucidate soft tissue regeneration [120]. Among the critical signaling pathways associated with epithelial stem cell function, phosphatidylinositol 3-kinase (PI3K)/Akt/mammalian target of rapamycin (mTOR) signaling has attracted extensive attention in soft tissue repair and regeneration [121, 122]. Akt activation occurs after Thr308 and Ser473 phosphorylation, and active Akt controls multiple cellular regulatory processes, including cell survival and cell metabolism [121, 123]. Akt activation is negatively regulated by molecules that antagonize PI3K signaling, whereas in vivo results have indicated that double-knockout of Akt1 and Akt2 leads to deficient activation of mTOR [124]. A prior study has indicated that ESCs provide protective mechanisms for inducing stem cell differentiation under the aberrant activation of mTOR [124]. Thus, strategies aimed at indirectly activating mTOR may be a feasible approach to increase epithelial migration into injured sites and wound beds, thereby promoting soft tissue regeneration.

Wnt/β-catenin signaling is a well-documented Wnt signaling pathway, and its roles in soft tissue homeostasis and regeneration have received ample interest in recent decades. A previous study has revealed that Wnt/β-catenin signaling is critically involved in the regulation of stem cell function and tissue repair, as well as in the progression of chronic inflammatory diseases [125, 126]. Within the nucleus, β-catenin binds T-cell factor transcription enhancers, thus promoting the transcription of specific genes and leading to specific Wnt/β-catenin transduction [127]. Prior studies have demonstrated that activation of this β-catenin-dependent pathway enhances the proliferation and function of stem cells, such as ESCs and MSCs, thus markedly promoting soft tissue regeneration [128–130]. Therefore, selective enhancement of Wnt/β-catenin signaling may be an effective strategy to induce soft tissue regeneration.

In addition, the role of nuclear factor erythroid 2-associated factor 2 (Nrf2) during soft tissue regeneration is an important research topic from the therapeutic perspective. Nrf2 is the primary mediator of active redox homeostasis. Several biofactors have been found to ameliorate cellular oxidative stress and enhance stem cell function, thus accelerating tissue repair by promoting Nrf2 activation [131]. The important role of Nrf2 in regeneration involves the prevention of reactive oxygen species (ROS) accumulation in damaged tissues and activation of the antioxidant defense system [132]. In a previous study, Nrf2 signaling has been demonstrated to have a protective role against cellular ROS via activation of the antioxidative system during tissue regeneration [132]. Excessive ROS suppresses the proliferation of stem cells, stimulates cell apoptosis, and impairs tissue regeneration [133]. Nrf2 is expressed in a wide array of cell types, including stem cells, endothelial cells, and fibroblasts. A recent study has indicated that Nrf2 deficiency impedes corneal epithelial wound healing in an Nrf2 knockout murine model [119]. Similarly, an in vivo study has indicated that Nrf2 deficiency inhibits the activation of the antioxidative system in keratinocytes [134]. Although these findings have indicated an important role of Nrf2 signaling during soft tissue repair and regeneration, more in-depth studies are needed to gain a better understanding of Nrf2 function in soft tissue regeneration. In Table 3, we summarize the crucial signaling pathways involved in soft tissue regeneration, each of which has been investigated to determine the underlying mechanisms in response to different microenvironment cues.

**Table 3:** Main signaling pathways in soft tissue regeneration

Signaling pathways	Major characteristics and functions	Applications in bone regeneration
JNKs	JNKs are known to be significant molecules mediating the intracellular response of stem cells to many different types of stimuli present in the external cellular microenvironment [115, 116]. JNK function is essential for building a delicate balance between cell death and stem cell survival in order to promote soft tissue repair, remodeling, and regeneration [117]	In a mouse wounding model, transplantation of preconditioned stem cells was capable of enhancing soft tissue regeneration with a robust antioxidant defensive mechanism through activation of JNKs signaling [118] Activation of JNK signaling promotes the differentiation of MSCs into keratinocytes and promotes tissue regeneration in mice [119, 120]
Akt	Akt activation occurs following Thr308 and Ser473 residues phosphorylation, and active Akt is capable of controlling multiple cellular regulatory processes, ranging from cell survival to cell metabolism [121–123]	Activation of PI3K/Akt/mTOR signaling enhances soft tissue repair and regeneration in the mouse wounding model [121, 122, 124]
Wnt	The role of Wnt/β-catenin signaling in soft tissue homeostasis and regeneration is well-documented. Wnt/β-catenin signaling is critically involved in the regulation of stem cell function and tissue repair, as well as in the progress of chronic inflammatory diseases [125, 126]. Within the nucleus, β-catenin binds to T-cell factor transcription enhancers, thus promoting the transcription of specific genes and a specific Wnt/β-catenin transduction outcome [127]	Activation of this β-catenin-dependent pathway can enhance the proliferation and function of stem cells such as ESCs and MSCs, markedly promoting soft tissue regeneration in mice [128–130]
Nrf2	Nrf2 is the primary mediator of active redox homeostasis, and it was previously found that some biofactors ameliorate cellular oxidative stress and enhance stem cell function, accelerating tissue repair by promoting Nrf2 activation [131]. The important role of Nrf2 in regeneration is the prevention of ROS accumulation in damaged tissues and activation of the antioxidant defense system [132]	In the mouse wounding model, Nrf2 signaling was demonstrated to act a protective role against cellular ROS via activation of the antioxidative system during tissue regeneration [133, 134] Nrf2 deficiency impedes corneal epithelial wound healing in a Nrf2 knockout (Nrf2-KO) murine model [119]
ERK1/2	Erk1/2 is activated by phosphorylation of various growth factors, ion rays, and hydrogen peroxide and affects the function of transcription factors such as c-MyC, c-FOS, c-Jun, ATF, NF-κB and AP-1. Erk1/2 promotes the transcription and expression of genes, which is closely related to cell proliferation and differentiation [135]	Visfatin could enhance wound repair and regeneration via activation of ERK1/2 signaling pathway in mice [136]
JAK	JAK/STAT signaling plays a prominent role in cellular stress, inflammatory response, and wound regeneration and regulates the physiological balance of the body [137]. This pathway is not only involved in cell proliferation, differentiation, and apoptosis but also related to immunomodulatory biological processes [138]	Local injection of JAK or STAT inhibitor could markedly delay wound repair and regeneration in mice [138]
HIF-1α	In diabetes, the suppression of HIF-1α could lead to a failure of the wound to activate VEGF in response to soft tissue ischemia, resulting in impaired wound angiogenesis and regeneration [139]	Injection of roxadustat is capable of accelerating diabetic wound healing via activating the HIF-1α/VEGF/VEGFR2 signaling in diabetic rat model [139]

*ESCs* embryonic stem cells, *JNKs* c-Jun N-terminal Kinases, *mTOR* mammalian target of rapamycin, *Nrf2* nuclear factor erythroid 2-associated factor 2, *ROS* reactive oxygen species, *STAT* signal transducer and activator of transcription, *VEGFR2* vascular endothelial growth factor receptor 2, *MSCs* mesenchymal stem cells, *ESCs* embryonic stem cells, *ROS* reactive oxygen species, *c-MyC* v-myc avian myelocytomatosis viral oncogene homolog, *c-FOS* serum response factor, *c-Jun* proto-oncogene, *ATF* activating transcription factor, *NF-κB* nuclear factor kappa-B, *AP-1* activator protein 1, *HIF-1α* hypoxia inducible factor-1α

## Immune microenvironment in tissue regeneration

The immune microenvironment plays a crucial role in tissue regeneration. Tissue regeneration generally begins with early immune-inflammatory responses, thus triggering the boosting of immune cells and secretion of inflammatory cytokines and chemokines, which subsequently mobilize and recruit immune cells to injured sites [140, 141]. Simultaneously, stem cells can cope with an immune microenvironment and regulate immune-inflammatory responses during tissue regeneration [141]. Therefore, we will discuss the immunomodulatory effects of various immune cells and their roles in tissue regeneration.

### The role of macrophages in tissue regeneration

**Fig. 1:**
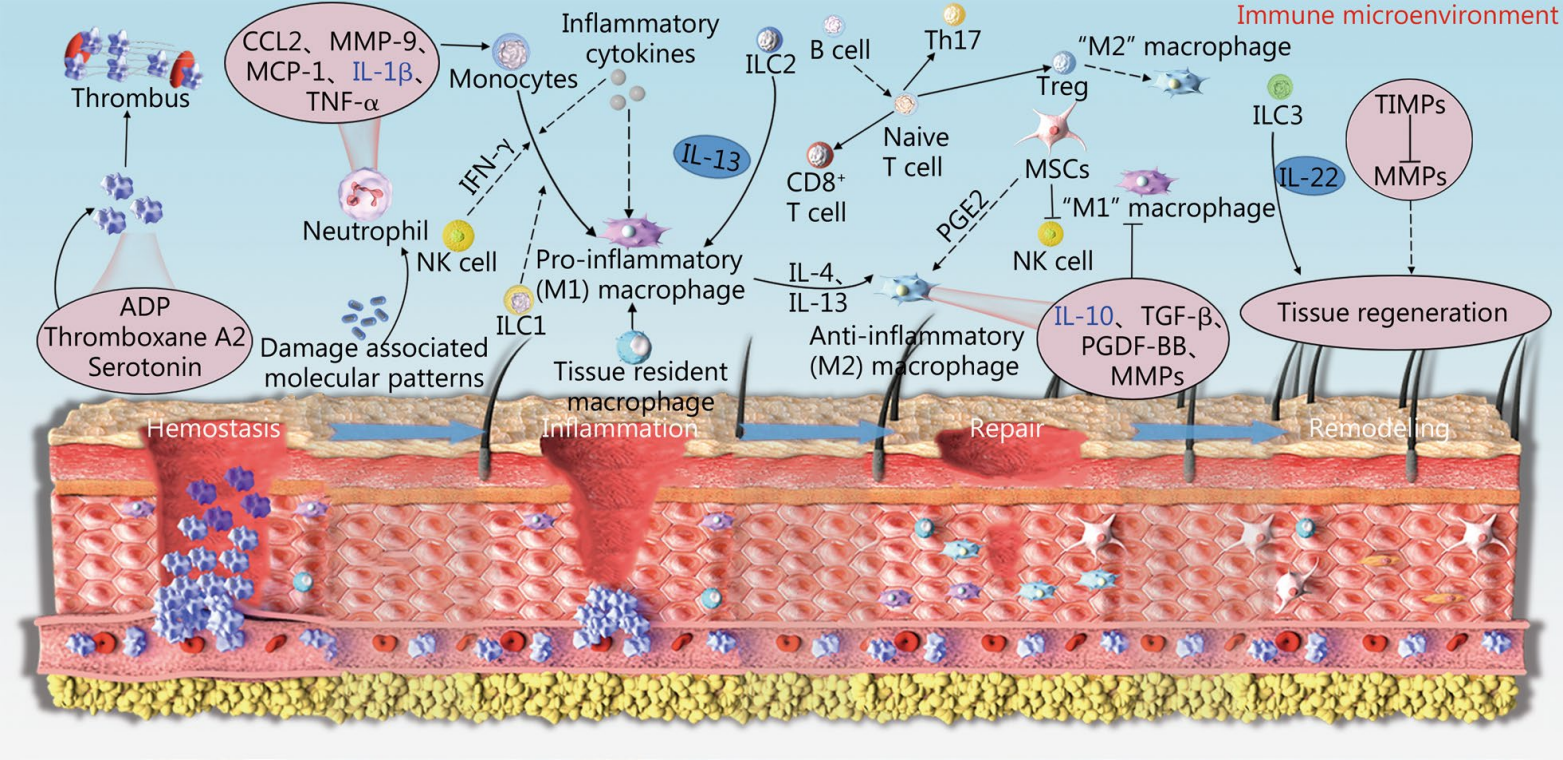
Important immune molecules and signaling during tissue regeneration. Four continuous and overlapping stages involved in tissue regeneration process, including hemostasis, inflammation, repair, and remodeling. These stages were tightly controlled and the development of these stages is dependent on the regulatory roles of immune cells, particularly at the inflammatory stage, thus determining the effectiveness of the subsequent repair and remodeling stages. MMPs matrix metalloproteinases, MSCs mesenchymal stem cells, NK natural killer, TGF-β transforming growth factor-β, TIMPs tissue inhibitor of metalloproteinases, CCL2 chemokine (C-C motif) ligand 2, MCP-1 monocyte chemoattractant protein-1, TNF-α tumor necrosis factor-α, IFN-γ interferon gamma, ILC1 unconventional NK cells, PGDF-BB platelet-derived growth factor BB

The process of tissue regeneration has been described as four continuous and overlapping stages: hemostasis, inflammation, repair, and remodeling [140]. To achieve an ideal outcome, these stages should be tightly controlled, because aberrations can lead to damage that increases the likelihood of regeneration failure. The development of these stages is dependent on the regulatory roles of immune cells, particularly at the inflammatory stage, thus determining the effectiveness of the subsequent repair and remodeling stages (Fig. 1) [140, 141].

For bone, cartilage, and soft tissue regeneration, inflammation initiates an influx of neutrophils, followed by monocytes, which then differentiate into macrophages [140]. Signals from innate immune cells further recruit lymphocytes into the wound bed, where they participate in intercellular communication and affect inflammatory responses. The next repair stage is characterized by neo-angiogenesis, secretion of ECM, and collagen synthesis. The ultimate remodeling stage involves maturation of the newly formed blood vessels and tissue remodeling [141]. Immune cells are crucial for the removal of cell debris and for modulating regeneration through the regulation of tissue-specific stem cells. This function is exemplified in bone repair and regeneration, in which the interaction between immune cells and osteogenic cells is critical for the completion of the inflammation stage and the progression to repair and remodeling [142]. In previous research, we have found that excessive inflammation hinders bone remodeling, whereas effective control of the inflammatory response induces bone regeneration [143]. Increasing evidence indicates that abnormal cellular activity during inflammation impairs tissue regeneration [144–146]. Given the complex roles of the immune microenvironment in tissue regeneration, in-depth knowledge of the underlying mechanisms that regulate tissue regeneration is essential.

Macrophages, an important immune cell type with multiple functions, have prominent roles in both innate and adaptive immunity. Macrophages extensively infiltrate damaged tissues and are key players in tissue regeneration [147]. In addition to their ability to eliminate cell debris, neutrophils, invading organisms, and other apoptotic cells through phagocytosis, macrophages actively mediate tissue repair and exhibit different phenotypes during tissue regeneration [147, 148]. M1 macrophages produce pro-inflammatory cytokines, are highly phagocytic, and can engulf apoptotic neutrophils and remove pathogens and debris from local tissues. M2 macrophages have anti-inflammatory effects, and regulate angiogenesis, fibroblast regeneration, myofibroblast differentiation, and collagen production [149]. Macrophages exert a crucial role in tissue regeneration through phenotypic polarization, and they participate in almost all stages of tissue regeneration. Several major cytokines are involved in regulating tissue regeneration via the mediation of macrophage phenotype polarization. Generally, IFN-γ, IL-2, IL-3, IL-12, tumor necrosis factor-α (TNF-α), lipopolysaccharide, and Toll-like receptor agonists induce macrophage M1 polarization [150–152]. M1 macrophages secrete pro-inflammatory cytokines and chemokines, such as IL-1β, IL-6, IL-12, IL-23, chemokine (C-X-C motif) ligand (CXCL)-9, and CXCL-10, and participate in inflammatory responses [153–155]. Accordingly, several cytokines, including IL-4, IL-10, IL-13, transforming growth factor-β (TGF-β), and granulocyte-macrophage colony-stimulating factor, induce macrophage M2 polarization and secretion of various anti-inflammatory molecules, thus enhancing anti-inflammatory activity and promoting tissue regeneration [156–158].

In early stages, macrophages infiltrate the wound area and are activated to the M1 phenotype, which participates in the phagocytosis of pathogens and cell fragments, and the secretion of inflammatory factors to recruit circulating monocytes. In the repair stage, macrophages produce active cytokines, thus promoting more apoptosis of neutrophils; elicit a switch from the pro-inflammatory phenotype (M1) to the anti-inflammatory phenotype (M2); and phagocytose apoptotic neutrophils, thereby alleviating local inflammation of the damaged tissue [159]. The pro-inflammatory ability of macrophages is important in the early stages of tissue regeneration, but proper tissue regeneration requires a timely transformation of macrophages to the anti-inflammatory phenotype.

The ability of macrophages to induce inflammatory deactivation is seen with the formation and maintenance of regulatory T cells (Tregs). Tregs create an anti-inflammatory microenvironment conducive to tissue regeneration and maintain the anti-inflammatory phenotype of macrophages [160]. Enhanced switching from a pro-inflammatory to an anti-inflammatory macrophage phenotype facilitates tissue regeneration. Scavenger receptor class B1 has been found to promote M1 macrophages switching to M2 macrophages for tissue regeneration [161]. Similarly, Kim et al. [162] have introduced an exosome-guided macrophage reprogramming technique, in which M2 macrophage-derived exosomes induce a complete switch from M1 to M2 macrophages, thereby markedly promoting cutaneous wound healing via enhancement of angiogenesis, re-epithelialization, and collagen deposition.

Different immune cells and immunomodulators are involved in the multiple stages of tissue regeneration. Tissue regeneration can be promoted by regulation of the immune system, particularly critical immune cell subsets [163]. However, the mechanism through which the immune system regulates the regeneration of various organs and tissues requires further study [164]. Macrophages are essential in most stages of tissue regeneration, but the mechanisms through which they switch their phenotypes and promote tissue regeneration remain elusive and require further exploration.

### The correlation between natural killer (NK) cells and tissue regeneration

NK cells are another important innate immune cell type recruited to sites of injury [165]. NK cells secrete active factors, which effectively mediate the host’s immune response. The key function of NK cells is to identify foreign, virally infected, and metabolically altered cells, and to induce their apoptosis or cell lysis [166]. The exact role of NK cells in the regulation of tissue regeneration remains unknown. NK cells have been well documented to remove injured cells at the site of damage, and the cytotoxicity of NK cells is regulated by an array of receptors and the distribution of ligands on the membranes of target cells [167]. Activated cytotoxic NK cells exert killing by delivering lytic granules or secreting death-inducing cytokines [168]. Dastagir et al. [169] have found that NK cells are recruited to regenerating digit tips, and have observed NK cytotoxicity against osteoclast and osteoblast progenitors. The authors have concluded that stem cell proliferation and differentiation are mediated through multiple routes by distinct NK cell subsets. In-depth knowledge of NK cell-stem cell crosstalk may provide novel strategies for regenerative medicine.

The interaction of NK cells and MSCs in regeneration has recently become an important research area. MSCs are trophoblasts that are likely to exist in all tissues to support the survival and growth of many cell types, including hematopoietic cells, ESCs, tumor cells, nerve cells, liver cells, and endothelial cells [170, 171]. The “cell empowerment” and “cell replacement” concepts describe how MSCs modulate the immune system and provide a source of undifferentiated cells for tissue regeneration [172]. Previous work has demonstrated that undifferentiated MSCs suppress NK cell proliferation, cytokine release, and cytotoxicity [173]. Interestingly, more recent evidence has suggested that in appropriate contexts, MSCs can also support NK cell function [174]. A prior study has further examined the crosstalk between MSCs and NK cells, and indicated that MSC activation enhances the regenerative functions of NK cells; moreover, NK cell-modulated neo-angiogenesis and tissue proliferation during trophoblast invasion are also used by MSCs in peripheral tissues to induce regeneration after inflammation [175]. However, MSCs have been demonstrated to impair the cytotoxic capabilities of NK cells [176]. Thus, more in-depth studies on the crosstalk between NK cells and MSCs are needed. The crucial issue in determining the outcome of using MSCs in clinical trials may be the interaction between implanted donor MSCs and recipient immune cells.

### Dendritic cells (DCs) in the regulation of tissue regeneration

DCs are antigen-presenting cells that are critical in orchestrating adaptive immune responses and tissue homeostasis [177–179]. DCs initiate T cell responses and link innate and adaptive immunity by directing T cell differentiation into effector lineages [177, 180]. Except for immediate antigen processing and presentation, DCs are involved in homeostasis and disease regulation through cytokine secretion and the shaping of peripheral tolerance through local immunity [181]. Although the precise role of DCs during tissue healing and regeneration remains under investigation, many studies have shown that DCs are fundamental in the tissue repair process. DCs recognize foreign substances at injury sites and immediately contribute to the healing of damaged tissue, acting as an immunoregulator of tissue regeneration through the modulation of macrophage homeostasis [182]. In a burn wound murine model, DC-deficient mice show significantly delayed wound healing associated with the inhibition of early cellular proliferation, wound levels of Transforming growth factor-β1 (TGF-β1), and neo-angiogenesis in the wound beds. These findings suggest that DCs may have an essential role in the acceleration of events that promote early wound healing, and this acceleration is likely to be caused by the secretion of factors that activate cell proliferation and enhance cell functions [183].

DCs interact with skeletal cells, which have critical roles in tissue repair and regeneration. MSCs inhibit DC maturation in vitro and impair the ability of DCs to prime T cells in vivo [184]. MSCs diminish major histocompatibility complex class II, CD40, and CD86 costimulatory molecules’ expression on mature DCs, and IL-6 is involved in the MSC-mediated immunoregulatory mechanism through partial inhibition of the differentiation of DCs [185]. MSC migration is promoted through EVs from DCs, which can be manipulated to locally recruit endogenous or transplanted cells to injury sites [186]. DCs have crucial roles in bone metabolism [187], including: 1) contribution to inflammation-mediated osteoclastogenesis and participation in inflammatory bone disease; 2) activation of T cells that produce bone remodeling cytokines and soluble factors; 3) pathogenesis of postmenopausal osteoporosis; 4) and transdifferentiation into osteoclasts in the presence of RANKL and macrophage colony-stimulating factor (M-CSF) or IL-17, wherein RANKL/RANK regulates the immune crosstalk between CD4 T cells and DCs. An in vitro study has indicated that DCs inhibit the differentiation and mineralization of osteoblasts [188]. The field of tissue engineering in regenerative medicine often relies on strategies to appropriately modulate the immune response. DCs directly interact with biomaterials and which are critical for exerting biomaterial function [180]. The crosstalk and mechanisms of regulation between bone cells and DCs must be further investigated before DCs can become a focus of new clinical therapies involving tissue regeneration and repair.

### T cells in tissue regeneration

**Fig. 2:**
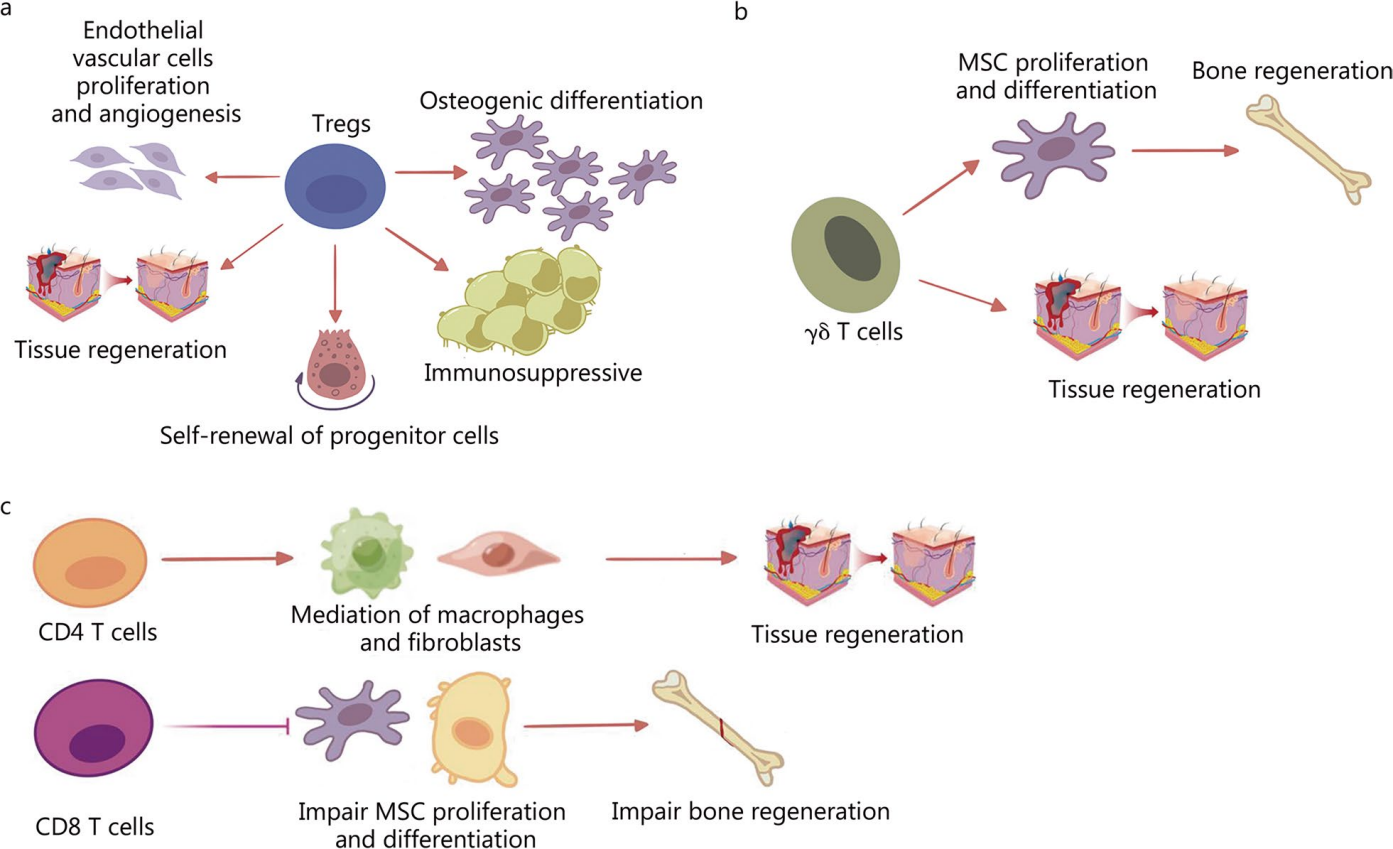
The important roles of T cells in the regulation of tissue regeneration. **a** Tregs modulate the activity of many other types of stem and progenitor cells involved in regeneration, and have become a crucial cell type for tissue repair and regeneration. **b** γδ T cells promote tissue repair and regeneration through communication with tissue stem cells. **c** CD4 T cells enhance tissue regeneration through the regulation of macrophages and fibroblasts, and CD8 T cells impair bone remodeling by hindering MSC proliferation and differentiation. MSCs mesenchymal stem cells

Tregs are an important group of T cells that maintain the body’s immune tolerance. Tregs are produced by the thymus and exported to the periphery. They actively inhibit the activity of potentially autoreactive T cells, thus modulating the body’s immunity and preventing the occurrence of autoimmune diseases [189]. Tregs are not only critical for immune homeostasis but also exert a variety of non-immune functions, including mediation of stem and progenitor cell activity. Consequently, Tregs have become a crucial cell type for tissue repair and regeneration [190]. In a prior study, we have demonstrated cross-communication between Tregs and bone-forming cells, with the potential for osteogenic differentiation and angiogenesis promoting bone remodeling and regeneration. Mechanistically, Treg-induced TGFBR1/SMAD2 signaling inhibition has been shown to be involved in the Tregs’ beneficial effects on bone healing [191]. Similarly, Tregs modulate the activity of many other types of stem and progenitor cells involved in regeneration. For example, enrichment in IL-33 derived from Tregs has been found to have a prominent role in the regulation of fibro/adipogenic progenitor cells, and diminished IL-33 is the main reason for failed tissue regeneration in aging mice [192]. Tregs systemically maintain the balance between immune homeostasis and inflammation, and are particularly abundant in soft tissue [193]. Tregs in soft tissue have also been found to enhance the regenerative process, mainly by enforcing immune tolerance and suppressing excessive inflammation [194]. For instance, Moreau et al. [195] have reported that Treg-derived amphiregulin induces tissue-resident T cell proliferation upon injury, thus leading to an immune-suppressive microenvironment and tissue regeneration (Fig. 2a).

Gamma delta (γδ) T cells are an important T cell type distributed in various tissues [196, 197]. Although the definitive effects of these cells on tissue regeneration are unknown, reports have identified the role of γδ T cells in promoting tissue regeneration, possibly through communication with tissue stem cells [198]. The involvement of γδ T cells in wound repair and regeneration has been determined on the basis of the dynamics of γδ T cells and γδ T cell-derived activators during tissue regeneration [199]. Similarly, the important role of γδ T cells has clearly been demonstrated in diabetic mice, which show delayed formation of granulation tissue and wound closure after γδ T cell ablation [200]. In bone regeneration, IL-17 A-producing γδ T cells promote bone regeneration by inducing osteogenesis in a fracture mouse model. Mechanistically, γδ T cells enhance the proliferation and osteogenic differentiation of injured MSCs, and consequently stimulate bone regeneration after injury [201] (Fig. 2b).

Other important T cell types, such as cytotoxic T cells (CD8 T cells) and T helper cells (CD4 T cells), have been well documented to be essential activators in tissue regeneration. Adoptive T-cell therapy is a promising therapeutic approach against diseases [202]. CD4 T cells play an important role in the immune system and guide the body to fight against microorganisms [203]. CD8 T cells, another subgroup of T cells that can be further activated into effective T cells, known as cytotoxic T lymphocytes, exist in the tonsils, spleen, and other organs [204]. A prior animal experiment has demonstrated that CD8 T cells impair cancellous bone repair [205]. Similar results have also been found in humans, thus suggesting that impaired bone regeneration is closely linked to the elevation of CD8 cells [206]. Interestingly, in skin regeneration, whereas CD4 and CD8 T cells are enriched in wounds, showing peak levels at days 5–10 and 7–10 post-injury, neither CD4 nor CD8 T cells are believed to impair skin regeneration [207]. Thus, CD8 and CD4 T cells may have different regulatory roles in the regenerative process, depending on the target tissue. In a rat model, in vivo, CD8 and CD4 T cells have opposing roles in mediating wound regeneration: CD4 T cells are associated with enhanced repair, whereas CD8 T cells are associated with impaired healing [208]. Mechanistically, T cells release a wide array of cytokines that affect both macrophages and fibroblasts, both of which play prominent roles in the regulation of tissue regeneration [209] (Fig. 2c).

## Roles of sEVs-derived immunomodulation in tissue regeneration

sEVs are nano-sized extracellular vesicles involved in the regulation of cell-to-cell communication, which have attracted attraction as a promising cell-free therapeutic strategy in clinical applications [210]. Generally, sEVs contain molecules including cytokines, lipids, and nucleic acids, which are important mediators of the biological behaviors of target cells [211]. sEVs derived from stem cells can achieve enhanced cell proliferation and function with little immune response by creating a beneficial immune microenvironment [212]. sEVs from MSCs have been demonstrated to enhance tissue regeneration and immune regulation, similarly to MSCs [213]. Thus, the regulatory roles of sEVs in tissue regeneration are discussed and summarized in the following section.

### sEVs-mediated immunomodulation and bone regeneration

In various models of diseases and cell-free regenerative medicine, sEVs appear to be beneficial in improving recovery [210, 211]. sEVs have been identified to play a major role in intercellular communication, particularly between MSCs and immune cells [212]. Tissue regeneration after injury requires two major conditions: 1) a pro-inflammatory microenvironment to neutralize injury and eradicate dead or injured tissue, and 2) a subsequent anti-inflammatory microenvironment to regenerate new tissue through the migration, differentiation, and proliferation of reparative cell types, thus increasing vascularization and nutrient supply [213].

MSC-mediated therapeutic activities are an important aspect of immune modulation, including MSC-derived sEVs [213, 214]. By comparing the immunomodulatory functions of human gingival mesenchymal stem cells (GMSCs) and GMSC-sEVs in a murine in vitro T cell co-culture model of collagen-induced arthritis, Tian et al. [215] have found that GMSC-sEVs have similar or sometimes greater effects than GMSCs in inhibiting IL-17 A and promoting IL-10, thus decreasing the frequency and intensity of bone erosion in arthritis; these findings suggest that GMSC-sEVs may be a promising new cell-free therapy strategy for treating rheumatoid arthritis. The sEVs collected from human Bone marrow-derived mesenchymal stem/stromal cells (hBMSCs) significantly decrease the expression of proinflammatory genes, such as *IL-1β*, *TNF‐α*, *IL‐6*, and inducible nitric oxide synthase (*iNOS*), in macrophages and greatly promote the expression of early osteogenic markers in hBMSCs, thus suggesting that sEVs derived from differentiating mesenchymal stem/stromal cells have a unique function in the regulation of bone dynamics through their osteoimmunomodulatory role [212]. An in vitro study has reported that T cell proliferation is suppressed by MSC-derived EVs, thereby supporting the application of a cell-based in vitro potency assay for determining the immunomodulatory potential of EVs [216]. Another in vitro experiment has characterized the immunomodulatory function of human adipose MSC-derived exosomes on in vitro stimulated T cells. The investigation confirmed that these exosomes repress the differentiation and activation of T cells, and decrease T cell proliferation and IFN-γ release in in vitro stimulated cells [217]. Yang et al. [218] have discovered that human umbilical cord MSC-derived exosomes released from hydrogels aid in bone regeneration in animal studies. Although this work had limitations regarding the overall mechanisms and efficacy, the strategy provides tremendous promise for prospective treatments in tissue and organ repair through sEV-based therapy. A recent porcine model study has identified MSC-derived sEVs paired with hyaluronic acid (HA) to aid in osteochondral repair by increasing trabecular bone thickness and improving the biomechanical properties of bone [219].

Immune cells affect bone regeneration through cell signaling regulation and osteoblastogenesis in MSCs. In an in vitro experiment, Li et al. [220] have identified that sEVs derived from M2 macrophages might have promise as a therapeutic tool in bone diseases, owing to their ability to inhibit adipogenesis and promote osteogenesis of BMSCs through the miR-690/IRS-1/TAZ axis. Our previous study has shown that hematopoietic cells stimulate proliferation and osteoblastogenesis, and inhibit cellular senescence of MSCs [221]. Hematopoietic cells express TNF-α, platelet-derived growth factor beta (PDGF-β), Wnt1, 4, 6, 7a and 10a, secreted frizzled-related protein 3 (sFRP-3), and sFRP-5. The increase of TNF-α expression in hematopoietic cells in older people is associated with activation of NF-κB signaling and/or Wnt/β-catenin signaling, which negatively affects the interactions of hematopoietic cells on MSCs via TNF-α receptors, through inducing cellular senescence while also inhibiting osteoblast differentiation in MSCs. Our data have established paracrine interactions of hematopoietic cells on human MSCs; these findings, together with those from other reports, suggest that declining skeletal stem cell function may involve the extrinsic mechanisms of immunosenescence [221–223]. sEVs play crucial roles in the cellular regulation of MSCs and immune cells, thus positively influencing bone regeneration. However, more studies are needed to precisely identify the mechanisms underlying exosomal immunomodulation and bilateral interactions between skeletal cells and immune cells in bone regeneration.

### Important roles of sEVs-mediated immunomodulation in regulation of cartilage regeneration

A variety of studies have examined the use of sEVs for cartilage regeneration. Notably, in these studies, the sEVs have been from an array of sources and applied at different concentrations, among which MSC-derived sEVs have been the most widely applied (Table 4). Previous studies have demonstrated that MSCs exert critical immunomodulatory roles in cartilage regeneration, mainly through paracrine secretion of trophic factors [224–226]. However, the immunomodulatory function of MSCs cannot be sufficiently imitated by any cytokine alone, e.g., IL-6, IL-8, IL-10, IL-33, monocyte chemoattractant protein-1 (MCP-1), or TGF-β, thus indicating that the immunomodulatory function of MSCs necessitates synergism among multiple cytokines [227, 228]. MSC-derived sEVs loaded with more than 100 immunomodulatory proteins are considered a perfect vehicle for this synergism [229]. A prior study has demonstrated that MSC-derived sEVs are immunomodulatory and not immunosuppressive in mice, and these sEVs induce Tregs with active immune reactivity triggered by the grafting of allogenic skin [230]. Furthermore, an in vivo study in an immunocompetent rat osteochondral defect model has indicated that MSC-derived sEVs alleviate OA by promoting M2 macrophage polarization and enhancing cartilage regeneration [230, 231]. MSC-derived sEVs are now widely accepted as a feasible therapeutic agent to regulate cartilage regeneration.

**Table 4:** Different sources of sEVs and their application in cartilage regeneration

Source	Cell experiment	Animal experiment	Utilization	Roles of sEVs in tissue regeneration	Potential mechanisms
Embryonic stem cell-derived MSCs	Chondrocytes; 5 µg/ml	Rat; dose: 2 mg/ml	Osteoarthritis treatment [232]	Promote temporomandibular joint repair and regeneration [232]	Ameliorate inflammation and suppress apoptosis and matrix degradation to achieve overall joint homeostasis [232]
MSCs	Primary chondrocytes; 10 µg/ml	–	Osteoarthritis treatment [233]	Promote chondrocytes proliferation [233]	Activation of miR-206/GIT1 axis [233]
Primary MSCs	Primary chondrocytes; 50 µg/ml	–	Osteoarthritis treatment [234]	Promote chondrogenesis and enhance chondrocytes proliferation [234]	Inhibit HDAC2/8 expression and promote cartilage matrix expression [234]
Embryonic stem cell-derived MSCs	-	Rat model; dose: 1 mg/ml	Cartilage regeneration [235]	Promote cartilage and subchondral bone repair [235]	–
Embryonic stem cell-derived MSCs	Primary chondrocytes; 0.1, 0.5 and 1.0 µg/ml	Rat model; dose: 1 mg/ml	Cartilage regeneration [236]	Promote chondrocytes proliferation and ameliorate inflammation [236]	Coordinated mobilization of multiple cell types and activation of several cellular processes [236]
MSCs	HUVECs; 100 µg/ml	–	Regeneration [237]	Promote angiogenesis [237]	Mediate stem cell-endothelial cell crosstalk [237]
Serum	MSCs, chondrocytes	–	Cartilage regeneration [238]	Promote MSCs towards chondrogenic differentiation [238]	Deliver sEVs-derived miR-140 to MSCs [238]

– No data. *SEVs* small extracellular vesicles, *MSCs* mesenchymal stem cells, *HDAC2* recombinant histone deacetylase 2, *HUVECs* human umbilical vein endothelial cells

Considerable developments have been achieved in the application of sEVs for cartilage regeneration, and the mechanisms, therapeutic strategies, and production have been widely studied [239]. In recent decades, clinical trials for sEVs have been undertaken, and standard guidelines for sEV extraction have been established [239]. Prior study has detected various bioactive molecules, including non-coding RNA, proteins, lipids, and cholesterin, in the content of sEVs [240]. However, full use of all characteristics of sEVs has not yet been accomplished, owing to the complexity of the bioactive cargo; most studies have focused on single bioactive factors within sEVs. An in-depth understanding of sEV biogenesis with the emergence of multiple bioreactors for elevating production would improve exosomal production in the laboratory setting, because the current low production scale greatly limits their clinical potential.

### sEVs regulate soft tissue regeneration via immunomodulation

Excessive and persistent inflammation after injury impairs soft tissue regeneration and leads to the formation of chronic tissue defects. Effective and rapid soft tissue regeneration can be achieved by suppression of the overactivity of immune cells at injury sites [241]. Normally, in the first few days after injury, immune cells, including macrophages, NK cells, and T cells, are recruited to the defect site by chemoattractants, such as complement, clotting components, and cytokines, to clear cell debris and bacteria from the wound. Thus, strategies that effectively modulate the overactive immune microenvironment have the potential to enhance and accelerate tissue regeneration [241].

Recent investigations have focused on identifying the mechanisms underlying the inhibitory effects of sEVs on the overactive immune response, including their suppressive activity on NK cells and CD8 T cells, their inhibitory effects on the differentiation and maturation of DCs, and their promotive effects on the function of Tregs [1, 242, 243]. Hence, sEVs are considered to have high potential as therapeutic vesicles for immunomodulation and for promoting soft tissue regeneration. For instance, Su et al. [241] have successfully engineered PD-L1-overexpressing sEVs, and demonstrated their promotive effects on wound healing through immunosuppressant activity.

Immunomodulation to ameliorate damage-induced inflammation and construct an appropriate immune microenvironment conducive to tissue regeneration may potentially be mediated through innate or adaptive immune responses [161]. Prior proteomic profiling research has revealed enrichment of exosomal proteins during inflammation or complement activation [242]. For example, MSC-derived sEVs have been reported to induce M2 phenotype polarization and decrease pro-inflammatory cytokines, thus enhancing tissue regeneration [243]. A crucial immunomodulatory advantage of sEVs allowing them to promote soft tissue regeneration involves the promotion of anti-inflammatory and pro-regenerative macrophages (M2) over pro-inflammatory macrophages (M1). Although the underlying mechanism has yet to be uncovered, the macrophage phenotype polarization observed in sEV-mediated tissue regeneration is attributable mainly to the ability of sEVs to communicate directly with monocytes, and modulate active molecule production and release. In addition to macrophages, Tregs have attracted attention for their link with sEVs in attenuating the activated immune system [236]. Interestingly, MSC-derived sEVs polarize CD4 T cells to Tregs when CD4 T cells are activated by allogenic CD11C^+^ antigen-presenting cells instead of CD3/CD28 co-stimulation [244]. This finding suggests that the extent of the immunosuppressive functions of sEVs depends on the immune-reactive microenvironment; consequently, exosomal immunomodulation may ameliorate the immune system without leading to adverse effects.

## Biomaterials involved in the regulation of the immune microenvironment for promoting regeneration

Biomaterials with different formulations have shown great promise in tissue regeneration [245]. The potential of immune microenvironment modulation by implanted biomaterials in vivo has attracted considerable attention, and the development of biomaterials for the regulation of immune responses is expected to promote tissue regeneration [246]. Microenvironment-regulating biomaterials may perform multiple functions in facilitating tissue regeneration. In the following section, advances in, and applications of, biomaterials used for tissue regeneration are discussed in depth.

### Biomaterial-based immunomodulation and its critical role in regulation of bone regeneration

A wide array of biomaterials have been identified as stabilizing structures for injured bone or inducers of bone regeneration. These differ in chemical composition, shape, porosity, and mechanical properties [245]. During the past few decades, extensive strides have been attempted to deliver immunomodulatory signals for bone regeneration through the use of a variety of biomaterials [246]. The “soft” biomaterials, represented by hydrogels, have structural and chemical properties that allow for: 1) dynamic changes in their mechanical characteristics; 2) processing into various forms with unique surface topographies; 3) activation of biological responses; and 4) sustained delivery of biofactors [247]. Our recent research has provided an example of how the bone immune microenvironment can be mimicked [109]; we developed a “cocktail therapy” to simultaneously regulate osteoblast differentiation and macrophage phenotype polarization. The cocktail therapy, comprising an HA-based hydrogel, engineered sEVs, and an inositol-requiring enzyme-1α (IRE-1α) inhibitor, has provided new insights into biomaterial strategies for effective bone regeneration therapy. Similarly, an in situ injectable hydrogel has been constructed via single-step equal volume mixing of a PBS solution of oxidized HA and hydrazide grafted gelatin, and its immunomodulatory function by the release of sEVs overexpressing PD-L1 in bone regeneration has been verified [143]. Additionally, with the rapid development of bio-nanotechnology, various nano-structured materials in soft materials, such as anisotropic nanoscale ligands [248] and composite nanoparticles [249], have also been applied to regulate cell behaviors and promote bone regeneration. For example, Wong et al. [250] have conjugated RGD-bearing magnetic nanoparticles (Fe_3_O_4_ coated with silica) to increase RGD tether mobility, which can be decreased through the application of an external magnetic field, thus increasing MSC adhesion, spreading, and osteogenic differentiation. Furthermore, a “self-regeneration” biomaterial concept has recently been proposed, whereby the promotion of vascularization and bone formation can be achieved without the need for introducing cells or other therapeutics [251]. This strategy is based on the presence of layered topographic cues, particularly in biomaterials that arrange the nanomorphologic cues into layered three-dimensional (3D) structures. A recent example of mimicking an extracellular tissue environment has been introduced by Hasani-Sadrabadi et al. [252], who have reported a novel periodontal membrane for guided tissue regeneration, which can mimic the complex extracellular environment of periodontal tissue and serve as functional tissue constructs for periodontal regeneration.

Among the immune cells in the bone microenvironment, macrophages have emerged as central to the immunomodulation of tissue regeneration. Particular focus has been placed on the role of phenotype switching, which remains controversial [253]. For instance, a recent study has revealed the utilization of a nanostructured polycaprolactone (PCL)/polyvinylpyrrolidone electrospun biomaterial in bone tissue regeneration via polarization of macrophages toward the M2 phenotype [254]. Generally, immunomodulation through biomaterials can be achieved via various strategies, including: 1) mediation of their physicochemical properties, 2) delivery of immunoregulatory activators, and 3) alteration of their mechanical properties [255]. For example, Lin et al. [256] have introduced a new application of sodium alginate hydrogels with different stiffness to mimic tissue repair in vivo and have found that MSCs in a stiffer matrix show faster migration than those in a softer matrix; this novel platform promotes MSC migration through mimicking the natural tissue repair process. In addition to the physicochemical properties, direct incorporation of immunoregulatory activators has been used to initiate a specific immune response and bone regeneration. In our prior work, we constructed mesoporous silica and FeO composite-targeted nanoparticles loaded with baicalein, which were capable of promoting bone regeneration via the delivery of active baicalein to injured bone sites [257]. Similarly, the ability of ECM proteins to promote M2 macrophage polarization has been used to enhance the immunomodulatory function of implanted biomaterials [258]. The emerging understanding of the role of the biomaterial-regulated immune microenvironment in bone regeneration has led to a paradigm shift in strategies that harness immune cells to promote regeneration.

### Immunomodulatory biomaterials for cartilage regeneration

Cartilage is susceptible to damage but cannot easily heal because of its avascular nature. Cartilage regeneration remains a research focus, because most currently used clinical methods cannot achieve satisfactory results [259]. Evidence indicates that synovial inflammation is closely associated with chondrocyte apoptosis and cartilage damage [260]. Macrophages reside in the synovial lining of joints and are involved in the regulation of synovial inflammation [261]. Furthermore, anti-inflammatory M2 macrophages have been proposed to produce pro-chondrogenic factors, such as TGF-β and insulin-like growth factor (IGF), which are considered novel targets for cartilage repair [262]. Therefore, innovative biomaterials scaffolds with immunomodulatory activity have been developed to promote cartilage regeneration in recent decades.

**Fig. 3:**
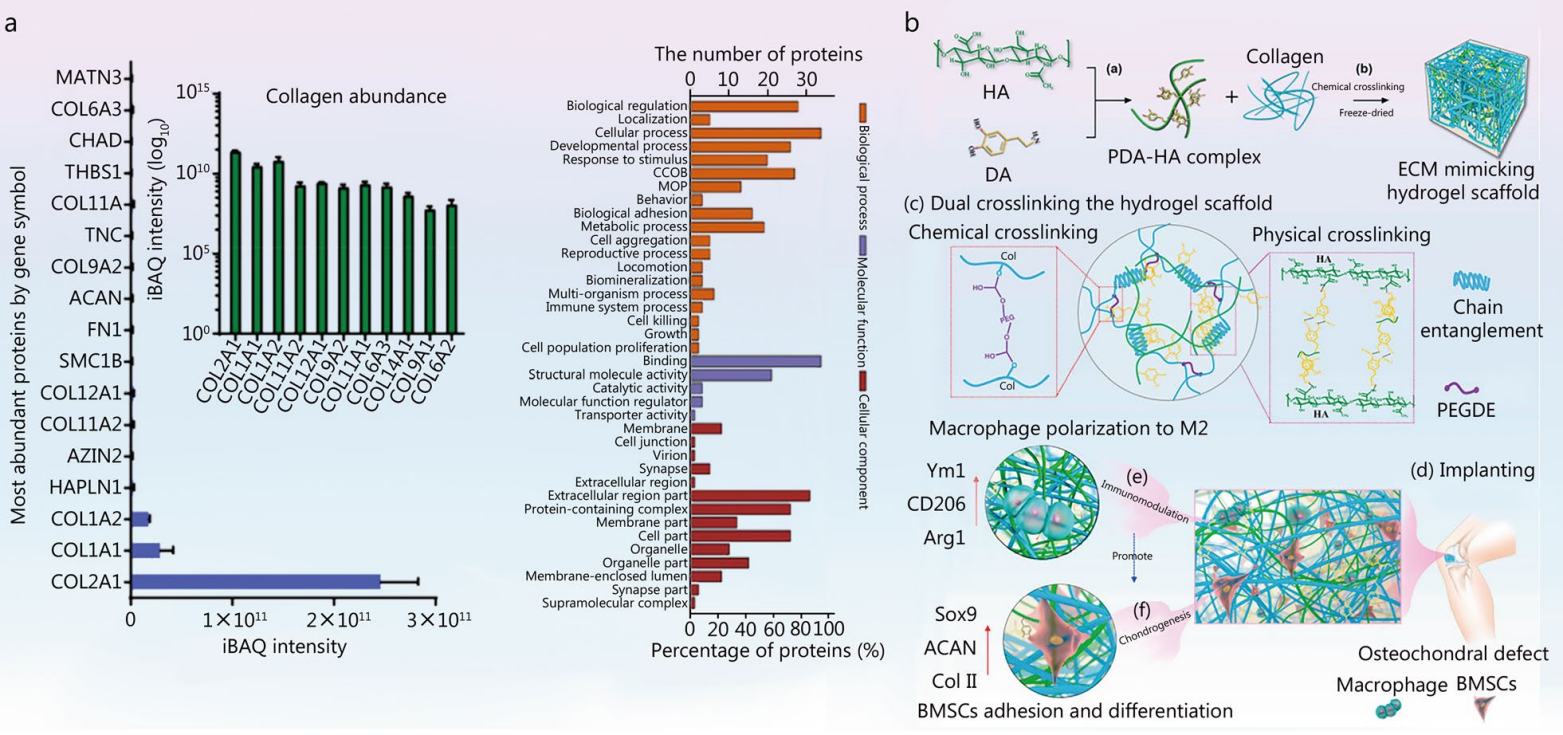
Biomaterial chemistry-based immunomodulation for cartilage regeneration. **a** Proteomic evaluation of decellularized cartilage ECM [266]. **b** Preparation of Col/PDA/HA hydrogel scaffold and therapeutic mechanism for cartilage regeneration [268]. Col collagen, ECM extracellular matrix, HA hyaluronic acid, PDA polydopamine, PEGDE polyethylene (glycol) Diacrylate, BMSCs bone marrow mesenchymal stem cells

Biomaterials derived from ECM have been reported to play important roles in modulating the host macrophage response [263]. Because of their ability to provide microenvironments similar to native cartilage, decellularized cartilage ECM scaffolds have been used to enhance cartilage regeneration [264, 265]. When implanted into cartilage defect areas, cartilage ECM scaffolds induce M2 phenotype polarization of macrophages, which in turn stimulates the migration, proliferation, and chondrogenic differentiation of MSCs [266] (Fig. 3a). Type II collagen, the main collagenous component in cartilage, is responsible for the induction of M2 macrophage polarization [262]. Another type of collagen, squid type II collagen, has been applied to repair cartilage lesions in degenerative OA [267]. This collagen polarizes the synovial macrophage response toward an M2 phenotype and increases the levels of TGF-β and IGF simultaneously in vivo, thus inducing a pro-chondrogenic environment for cartilage repair. An ECM-mimicking hydrogel scaffold has been fabricated by incorporating polydopamine (PDA)-modified HA into a collagen matrix [268] (Fig. 3b). The hydrogel scaffold has immunomodulation ability, through increasing the ratio of M2 macrophages and suppressing the inflammatory response, thus resulting in cartilage regeneration and remodeling in rabbits. Other natural biopolymers have also been suggested to have immunomodulatory functions. A hybrid scaffold composed of alginate, chitosan, hydroxyapatite, and fucoidan has an anti-inflammatory function, as evidenced by inhibition of the production of ROS and inflammatory mediators, including TNF-α, IL-6, and IL-1β, in lipopolysaccharide (LPS)-treated RAW 264.7 cells; thus, this scaffold should be a promising candidate for cartilage tissue engineering [269]. The immunomodulatory effects of some synthetic polymers have also been explored: 3D-printed porous scaffolds made of sulfonated polyetheretherketone have been demonstrated to facilitate cartilage repair by promoting M2 macrophage polarization, increasing secretion of the anti-inflammatory cytokines IL-4 and IL-10, and preventing macrophage-induced cartilage degeneration [270]. Additionally, lithium calcium silicate-based bioactive ceramic scaffolds have been reported to promote cartilage maturation by immunomodulating M2 macrophage polarization, as shown by upregulated expression of IL-10, and downregulated expression of the inflammatory factors TNFα, IL-6, and IL-1β [271].

**Fig. 4:**
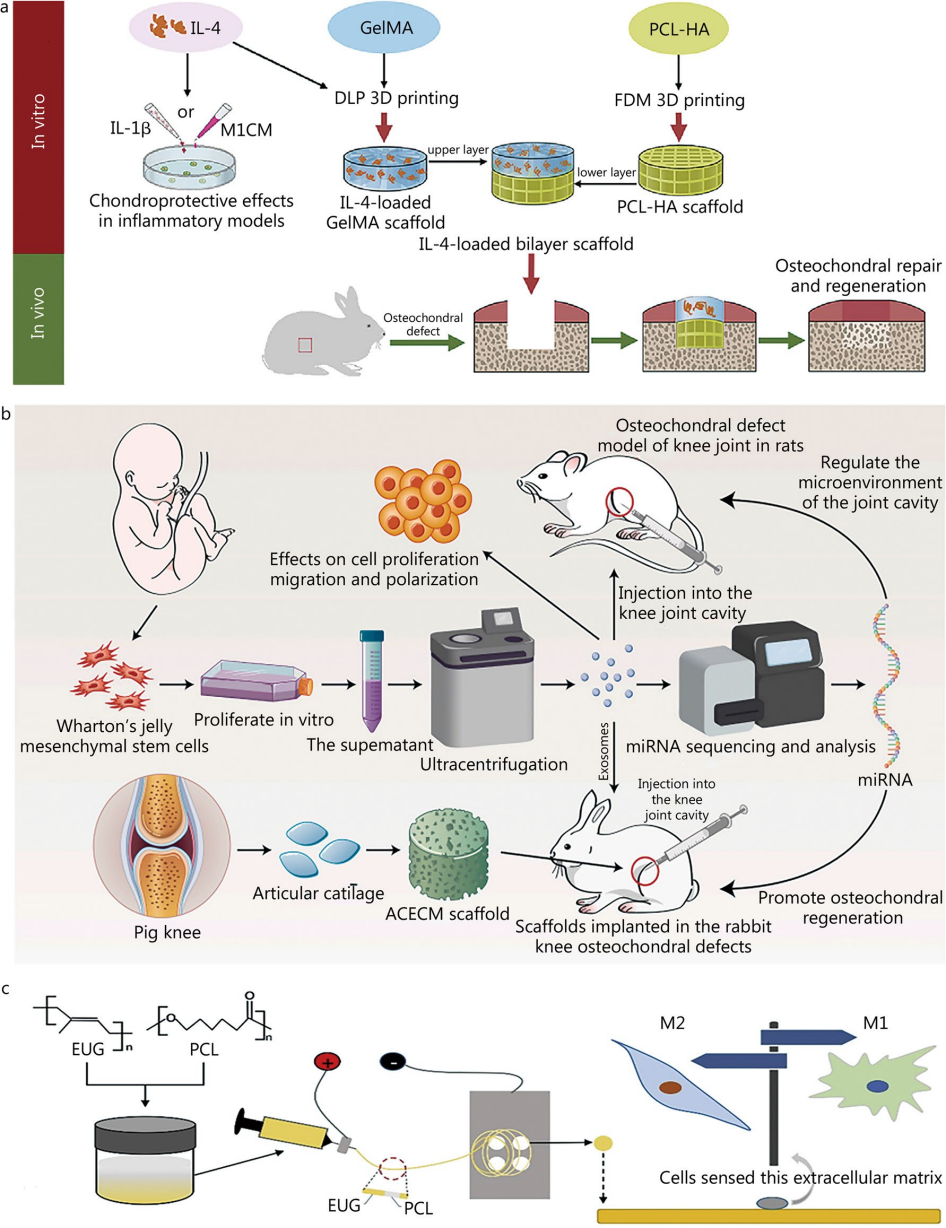
Biomaterial-based delivery system and physical property-based immunomodulation for cartilage regeneration. **a** Schematic representation of an IL-4-loaded bi-layer 3D printed scaffold for osteochondral regeneration [273]. **b** Schematic illustration of a cartilage ECM scaffold combined with Wharton’s jelly mesenchymal stem cell-derived sEVs for osteochondral regeneration [278]. **c** Effects of PCL/EUG scaffolds with different stiffness (akin to normal/osteoarthritic cartilage) on macrophage secretion behavior, adapted with permission from ref. [280], Elsevier. ECM extracellular matrix, EUG *Eucommia ulmoides* gum, PCL polycaprolactone, M1CM m1 macrophage conditional medium, DLP digital light projector, HA hyaluronic acids, FDM fused deposition modeling, ACECM acellular cartilage extracellular matrix

IL-4, a Th2-type cytokine, is an important promoter of M2 macrophage polarization [272]. Thus, delivery of IL-4 by bioscaffolds is an effective approach to drive macrophage polarization toward the M2 phenotype in vivo. An IL-4-loaded bilayer scaffold made of gelatin methacrylate (GelMA) (upper layer) and PCL-hydroxyapatite (lower layer) has been developed for the repair of osteochondral defects in rabbits [273] (Fig. 4a). IL-4 released from the scaffold relieves the inflammatory response and protects chondrocytes, thus improving regeneration of both cartilage and subchondral bone. Platelet-rich plasma has also been found to facilitate M2 macrophage transition. The incorporation of 20% platelet-rich plasma into GelMA hydrogel scaffolds enhances osteochondral repair effects in rabbits by promoting the proliferation, migration, and chondrogenic differentiation of BMSCs, and increasing M2 macrophage infiltration [274]. Recently, MSCs and their sEVs have been shown to have unique immunomodulatory properties and anti-inflammatory abilities [275, 276]. Bioscaffolds loaded with MSCs or their sEVs have been applied in the treatment of cartilage damage. BMSC-based engineered cartilage, produced by culturing BMSCs on PGA/PLA scaffolds, has been reported to suppress in vivo inflammation through the promotion of M2 macrophage polarization, thus improving cartilage regeneration [277]. In a rabbit articular cartilage defect model, the intra-articular injection of human umbilical cord Wharton’s jelly MSC-derived sEVs has been found to increase the infiltration of regenerative M2 macrophages and the expression level of IL-10, and to decrease the ratio of M1 macrophages, thus improving cartilage repair [278] (Fig. 4b). Additionally, a biocomposite scaffold composed of ECM/GelMA/sEVs has been reported to increase cartilage regeneration by promoting chondrocyte migration, restoring chondrocyte mitochondrial dysfunction, and enhancing the polarization of macrophages toward the M2 phenotype [279].

The physical properties of the scaffolds are also involved in the regulation of macrophage polarization and cartilage regeneration. PCL/*Eucommia ulmoides* gum composite scaffolds with a different elastic modulus that overlaps with those of human cartilage tissues with OA (1–5 MPa) have been designed by adjusting the ratio of PCL to *Eucommia ulmoides* gum. In this range, high scaffold stiffness favors M2 macrophage polarization, and the expression of inflammatory cytokines increases as the scaffold stiffness decreases [280] (Fig. 4c). However, the mechanism through which scaffold stiffness regulates the immune microenvironment and macrophage behaviors remain to be elucidated. Macrophages can sense mechanical stimulation via integrins, which produce signals to focal adhesion kinases and enhance cytoskeleton reorganization. Cha et al. [281] have revealed that macrophage polarization within 3D biomaterials can be modulated through integrin-mediated interactions. Inhibition of integrin α2β1 significantly decreases the induction of M2 macrophages. Kang et al. [282] demonstrated that highly anisotropic ligand-coated gold nanorods facilitate the recruitment of integrin β1 on macrophages, thus enhancing cell adhesion and M2 polarization. In addition, the effects of other scaffold properties, such as porosity, pore size, topography, and hydrophilicity, on macrophage behavior and cartilage regeneration deserve further investigation.

### Critical role of immunomodulation in biomaterials-mediated soft tissue regeneration

**Fig. 5:**
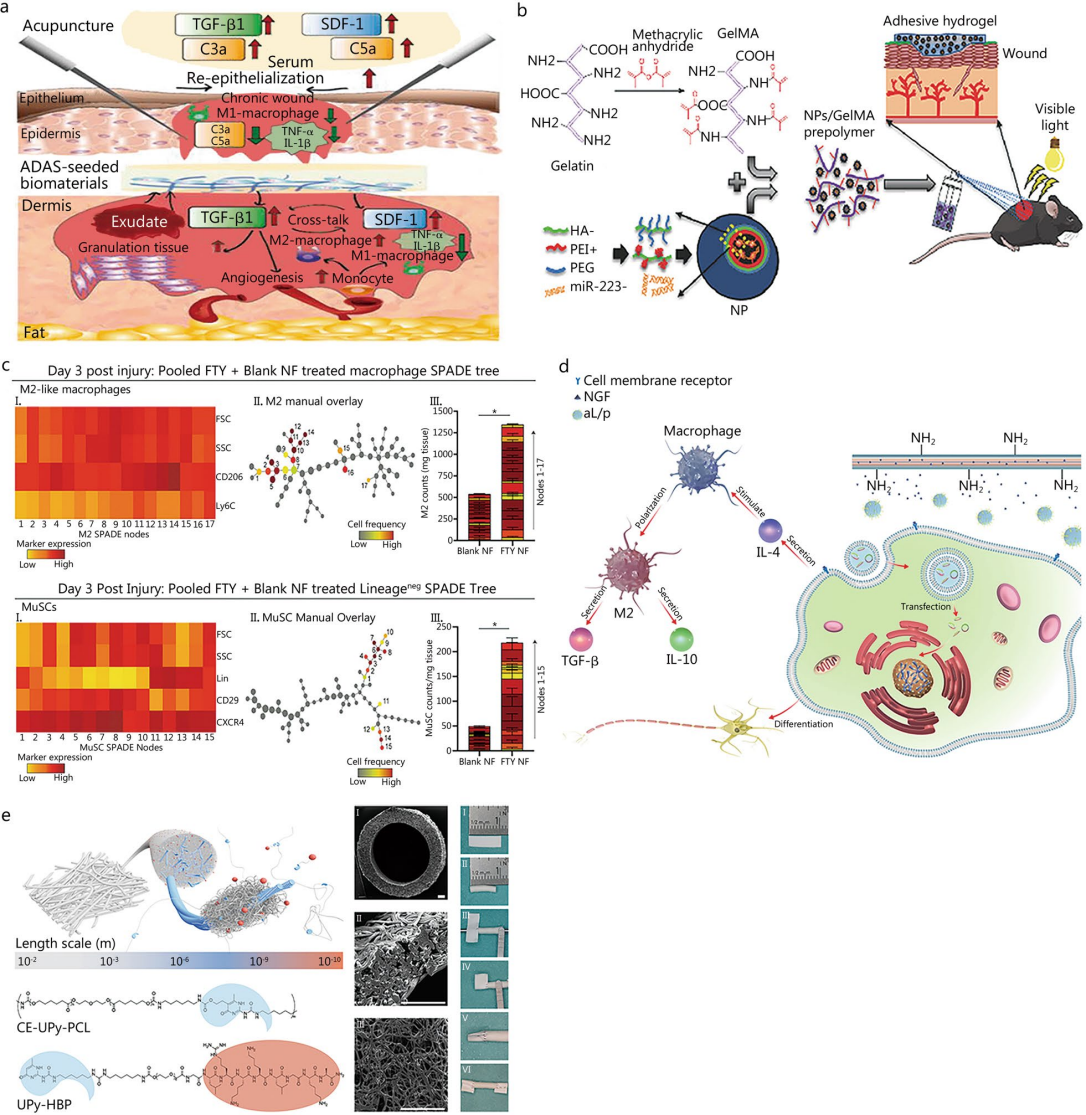
Biomaterial-based immunomodulation for soft tissue regeneration. **a** Schematic illustration of ADSCs-seeded chitosan/difunctional polyurethane hydrogel for the treatment of chronic diabetic skin wounds, adapted with permission from ref. [289]. **b** The hybrid hydrogel loaded with miR-223-laden nanoparticles promotes wound healing through increased M2 macrophage polarization, adapted with permission from ref. [290], Wiley online library. **c** Pseudotime analysis of the FTY720-induced increase in immune cell infiltration into a muscle defect area 3 days post-VML injury [291]. Copyright 2018, Elsevier. **d** Preparation of PLA electrospun fibers combined with pH-responsive IL-4 plasmid-loaded liposomes for the treatment of acute spinal cord injury. Copyright 2020, Nature Publishing Group [292]. **e** Development of an electrospun UPy-PCL scaffold functionalized with IL-4 and heparin for vascular damage repair [293]. Copyright 2021, Wiley online library. ADSCs adipose tissue-derived mesenchymal stem cells, PCL polycaprolactone, PLA polylactic acid, UPy ureido-pyrimidinone, VML volumetric muscle loss

The skin, the largest organ of the human body, is susceptible to multiple insults including accidental injuries and various diseases. The skin is equipped with an intricate network of immune cells, mainly including neutrophils, lymphocytes, monocytes, and macrophages, which is crucial not only for host defense but also for tissue homeostasis and reconstruction [283]. In the event of injury, a cascade of biological interactions between different cell types (immune cells, fibroblasts, endotheliocytes, and keratinocytes) and ECM components is initiated, thus inducing wound healing and tissue regeneration. The development of biomaterials for the regulation of immune responses will facilitate wound healing and tissue restoration. Naturally, derived biopolymer-based hydrogels have been found to have intrinsic anti-inflammatory activity [284]. Chitosan is a widely used biomaterial for wound healing, owing to its favorable biocompatibility, biodegradability, adhesiveness, and hemostatic ability [285]. A chitosan/aloe vera nanohydrogel, developed to enhance wound healing, has been demonstrated to decrease the ratio of M1 macrophages and the expression of iNOS and TNF-α while increasing the ratio of M2 macrophages, thereby promoting skin tissue regeneration [286]. Recently, the immunomodulatory function of silk fibroin has drawn the attention of researchers. Silk fibroin hydrogels applied in burn wound treatment have been found to induce a transition from the inflammation to proliferation stage, and improve tissue regeneration, as evidenced by the deposition of collagen type I and III fibers [287]. Interestingly, synthetic Ti_3_C_2_ MXene quantum dots have been reported to modulate the immune microenvironment by selectively inhibiting the activation of proinflammatory CD4^+^IFN-γ^+^ T cells while promoting the proliferation of immunosuppressive CD4^+^CD25^+^FoxP3^+^ T cells, thus enhancing wound healing in rats [286]. Although the immunomodulatory activity of hydrogels has been obtained through composition selection, their effectiveness is considered limited. Local delivery of MSCs, microRNAs (miRNAs), and biological molecules, such as anti-inflammatory cytokines (IL-10, IL-4, IL-2, and MCP-1), peptides (L-12 and LL-37 peptides), and antibodies (anti-TNF-α), has gradually become a common strategy for the regulation of the immune microenvironment at wound sites [284]. For example, an adipose tissue-derived MSCs (ADSCs)-seeded chitosan/difunctional polyurethane hydrogel has been prepared for the treatment of chronic diabetic skin wounds [288]. The hydrogel produces synergistic immunomodulatory effects through activation of C3a and C5a, upregulation of the cytokines stromal cell derived factor 1 (SDF-1) and TGF-β, and decreased secretion of the proinflammatory cytokines TNF-α and IL-1β, thus accelerating wound healing [289] (Fig. 5a). MiR-223 has recently been suggested to promote M2 macrophage polarization. An adhesive hydrogel containing miR-223-loaded HA nanoparticles has been reported to achieve local delivery of miR-223 and drive the polarization of macrophages to the M2 phenotype, thereby promoting wound healing and the formation of uniform vascularized skin [290] (Fig. 5b).

Muscle regeneration is also significantly affected by the activities of various immune cells, such as macrophages, CD8 T cells, and Tregs [294]. The immune-mediated muscle regeneration driven by scaffolds holds great promise in muscle regeneration. How skeletal- and cardiac muscle-derived ECM scaffolds regulate the immune microenvironment and stimulate tissue recovery in traumatic muscle wounds has been investigated [295, 296]. The ECM scaffolds induce IL-4-dependent M2 macrophage polarization through activation of the mTOR/Rictor-dependent T helper 2 pathway, thus guiding a pro-regenerative response that facilitates muscle regeneration. The effects of local delivery of RK35, a myostatin inhibitor, by HA/muscle-derived ECM scaffolds on muscle regeneration have been investigated [291] (Fig. 5c). The scaffolds show a prolonged release of RK35, thereby promoting pro-regenerative M2 macrophages and FoxP3^+^ Tregs, and increasing anti-inflammatory cytokine expression. In a recent study, PLGA/PCL electrospun nanofiber scaffolds loaded with FTY720, an agonist of the sphingosine-1-phosphate signal, have been demonstrated to promote pro-regenerative local injury milieu formation, as shown by increased numbers of M2 macrophages and muscle stem cells, thus improving muscle regeneration after volumetric loss [291].

Peripheral nerve regeneration remains a challenge, because the currently used autogenous tissue replacement is limited by tissue availability, secondary deformities, and potentially inappropriate size. To overcome these obstacles, various biomaterial scaffolds have been developed to repair nerve injuries in recent years. Hydrogel-based codelivery of MSCs and bioactive factors has also been applied for the treatment of nerve injuries. Fibrin hydrogels loaded with ADSCs and microspheres containing tacrolimus have been reported to enhance peripheral nerve regeneration via immunomodulatory actions [297]. In another study, on the basis of the acidic microenvironment at injury sites, pH-responsive IL-4 plasmid-loaded liposomes have been incorporated into PLA electrospun fibers to treat acute spinal cord injury (SCI) [292] (Fig. 5d). The immunoregulatory fiber scaffolds significantly suppress the acute inflammatory response and promote neural differentiation of MSCs, thus decreasing scar tissue formation and enhancing motor function recovery. However, a growing number of studies have demonstrated that the delivery of MSCs has only limited benefits for SCI, possibly because of the heterogeneity of MSCs [298]. Furthermore, some scaffolds negatively affect spine cord regeneration because of the proinflammatory milieu induced by biomaterials [299]. Thus, a more comprehensive understanding of the crosstalk among the immune system, implanted scaffolds or MSCs, and the nervous system will be essential for treating SCI. Immunomodulatory effects driven by biomaterial scaffolds are also an innovative regenerative strategy to repair vessels. Recently, an electrospun chain-extended-ureido-pyrimidinone-PCL scaffold functionalized with IL-4 and heparin has been developed to repair vascular damage in rats. The addition of IL-4 ameliorates the intimal hyperplasia caused by heparin and promotes M2 macrophage polarization and mature neotissue formation [293] (Fig. 5e).

## Conclusions

The immune system is closely associated with tissue injury and regeneration. Therefore, effective modulation and manipulation of the immune response to effectively modulate the innate healing process are crucial for successful tissue restoration. Spatiotemporal regulation of immune cells, their functions, and their communication with tissue-specific cells, including progenitor cells and stem cells, is imperative to enhance tissue regeneration. An in-depth understanding of the immunomodulatory and pro-regenerative activators and their multiple functions will critically contribute to their successful application as therapeutics.

Although much knowledge has been gained regarding immunomodulation and applied to the rational design of strategies to modulate the immune response and promote tissue regeneration, multiple underlying mechanisms remain to be explored. For example, M2 phenotype macrophages are widely accepted to be permissive to tissue repair and regeneration. During the inflammation phase, however, excessive infiltration of M2 macrophages is not conducive to tissue resistance against foreign pathogens and may thus impair tissue healing. The mechanisms underlying this dual function are not well described. Furthermore, other immune cells have subpopulations, and different subpopulations may exert different effects on tissue regeneration. For example, whereas CD8 T cells have adverse effects on tissue regeneration, CD4 T cells and Tregs enhance tissue regeneration. With the development of research technologies such as single-cell sequencing technology, further definitive classification of immune cell subpopulations will be beneficial in the study of the regulation of the immune microenvironment in tissue regeneration. In addition, immune regulation is a complex and delicate dynamic regulatory process. With aging, the function of the immune system gradually declines. Therefore, whether diminished tissue regeneration in older people is closely associated with the functional decline in the immune system must be investigated. An in-depth exploration of the underlying mechanisms would enable the immune system to be effectively harnessed to improve tissue repair.

The incorporation of biomaterials into immunomodulatory therapeutics has significant potential to advance the fields of tissue engineering and regenerative medicine. However, although many biomaterials that enhance tissue regeneration and mediate immunomodulation have been developed, most of the research has yet to be translated into the clinic. To enable the clinical utilization of biomaterials, materials with high biosafety profiles should be selected. Notably, precise targeting and controlled release are two issues requiring attention. Optimization of these two aspects should allow biomaterials to achieve precise regulation of the immune microenvironment and facilitate their clinical translation. Therefore, novel strategies that promote precise and targeted immunomodulation for clinical use in tissue are required.

## Data Availability

The datasets used or analyzed during the current study are available from the corresponding author on reasonable request.

## Abbreviations

ADSCs: Adipose tissue-derived MSCs
ASCs: Adult stem cells
AT-MSCs: Adipose tissue derived-MSCs
BM-MSCs: Bone marrow derived-MSCs
BMP-2: Bone morphogenetic protein-2
Col: Collagen
CXCL: Chemokine (C-X-C motif) ligand
DCs: Dendritic cells
ECM: Extracellular matrix
ESCs: Embryonic stem cells
EUG: *Eucommia ulmoides* gum
EVs: Extracellular vesicles
FAK: Focal adhesion kinase
FGF: Fibroblast growth factor
γδT cells: Gamma delta T cells
GelMA: Gelatin methacrylate
GMSCs: Gingival mesenchymal stem cells
HA: Hyaluronic acid
hBMSCs: Human bone marrow-derived mesenchymal stem/stromal cells
IGF: Insulin-like growth factor
iPSCs: Induced pluripotent stem cells
IL: Interleukin
IFN-γ: Interferon-γ
iNOS: Inducible nitric oxide synthase
IRE-1α: Inositol-requiring enzyme-1α
JNK: c-Jun N-terminal kinase
LPS: lipopolysaccharide
MCP-1: Monocyte chemoattractant protein-1
M-CSF: Macrophage colony stimulating factor
mTOR: Mammalian target of rapamycin
miRNAs: MicroRNAs
MSCs: Mesenchymal stem cells
NK cells: Natural killer cells
Nrf2: Nuclear factor erythroid 2-associated factor 2
OA: Osteoarthritis
PCL: Polycaprolactone
PDA: Polydopamine
PDGF-β: Platelet-derived growth factor beta
PI3K: Phosphatidylinositol 3-kinase
PGE2: Prostaglandin E2
rhBMP-2: Recombinant human BMP-2
ROS: Reactive oxygen species
SCI: Spinal cord injury
SDF-1: stromal cell derived factor 1
sEVs: Small extracellular vesicles
sFRP-3: Secreted frizzled-related protein 3
STAT: Signal transducer and activator of transcription
TGF-β: Transforming growth factor-β
TGF-β1: Transforming growth factor-β1
TMJ: Temporomandibular joint
Tregs: Regulatory T cells
TNF-α: Tumor necrosis factor-α
UPy: Ureido-pyrimidinone
VEGF: Vascular endothelial growth factor
VML: Volumetric muscle loss
